# Diagnostic testing for Legionnaires’ disease

**DOI:** 10.1186/s12941-017-0229-6

**Published:** 2017-08-29

**Authors:** David M. Pierre, Julianne Baron, Victor L. Yu, Janet E. Stout

**Affiliations:** 1 Special Pathogens Laboratory, 1401 Forbes Avenue, Pittsburgh, PA 15219 USA; 2 University of Pittsbrugh, Pittsburgh, Pennsylvania 15219 United States; 3 Department of Medicine, School of Medicine, University of Pittsburgh, Pittsburgh, PA USA

## Abstract

Legionnaires’ disease is commonly diagnosed clinically using a urinary antigen test. The urinary antigen test is highly accurate for *L. pneumophila* serogroup 1, however other diagnostic tests should also be utilized in conjunction with the urinary antigen as many other *Legionella* species and serogroups are pathogenic. Culturing of patient specimens remains the gold standard for diagnosis of Legionnaires’ disease. Selective media, BYCE with the addition of antibiotics, allows for a high sensitivity and specificity. Culturing can identify all species and serogroups of *Legionella.* A major benefit of culturing is that it provides the recovery of a patient isolate, which can be used to find an environmental match. Other diagnostic tests, including DFA and molecular tests such as PCR and LAMP, are useful tests to supplement culturing. Molecular tests provide much more rapid results in comparison to culture, however these tests should not be a primary diagnostic tool given their lower sensitivity and specificity in comparison to culturing. It is recommended that all laboratories develop the ability to culture patient specimens in-house with the selective media.

## Background

*Legionella* species are aerobic, intracellular, gram negative bacteria [1]. The genus encompasses more than 50 species and 70 serogroups; almost 50% of the species have been associated with disease in humans [1]. The species that causes over 90% of human disease is *L. pneumophila*, with serogroups 1, 4, and 6 being the most frequent serogroup [1]. *Legionella* can cause two distinct types of disease: Pontiac Fever and Legionnaires’ disease, a bacterial pneumonia. Among the 20,000–30,000 cases of Legionnaires’ disease reported annually, approximately 25% are hospital acquired [1]. More accurate estimates suggest that 56,000–113,000 cases occur in the US annually and most are not diagnosed (Edelstein P, private communication) [2]. The incubation period is 2–14 days [1]. High fever >39.5 °C, confusion and stupor, and multi-system organ failure (especially renal dysfunction) occur in the late stages of pneumonia. Gastrointestinal symptoms including diarrhea seem to be more common for patients with Legionnaires’ disease. The case fatality rate of healthcare-associated Legionnaires’ disease ranges from 38 to 53%, while community acquired disease carries an approximately 20% fatality rate. Low index of suspicion of by physicians for this pneumonia is likely the most important predisposing factor since highly effective antibiotic therapy exists. The average length of hospital stay is 10.3 days but ranges from 1 to 84 days, with a total of 13,000 patients hospitalized due to the disease per year [3]. It is estimated that the total cost of each case of Legionnaires’ disease per patient exceeds $34,000 and that the total cost of all hospitalizations is over $433,000,000 [3, 4].

### Urine antigen test for Legionella

Currently 97% of clinical diagnoses are obtained using a urinary antigen test [5]. These tests use monoclonal antibodies that specifically recognize most *L. pneumophila* serogroup 1 lipopolysaccharide antigens; they however, fail to detect disease caused by other serogroups of *L. pneumophila* or other species of *Legionella*. *L. pneumophila* serogroup 1 causes from 50 to 80% of Legionnaires’ disease; so as many as 20–50% of cases of Legionnaires’ disease remain undiagnosed if the urine antigen is used as the sole test for diagnosis [1, 5]. Other serogroups of *L. pneumophila* and other species are also important in disease, especially serogroups 4 and 6, and the species *L. micdadei* and *L. longbeachae* [6]. Approximately 8% of patients with Legionnaires’ disease do not excrete antigen in their urine [7]. The sensitivity and specificity range from 69 to 100% and 99 to 100% respectively [8–10]. Test results can be available within minutes following processing. Despite its weaknesses, the urine antigen test has revolutionized the diagnosis of Legionnaires’ disease given the ease of its performance and rapidity of the test.

### Culture of respiratory tract and environmental source

Culturing of patient specimens remains the gold standard for diagnoses of Legionnaires’ disease. Culture can identify all of the known *Legionella* species and serogroups. All known serogroups and species can be identified with culture. We found that the sensitivity for culture on selective media was 81% if culture was used as the gold standard [11].

Procedures for isolation of *Legionella* from patient specimens are not suitable for isolation from water sources. Buffered charcoal yeast extract (BCYE) is a media that was specifically formulated for the isolation of Legionella. In addition to BCYE, there are two other formulations of the BCYE agar for clinical isolation:

1. BYCE supplemented with polymyxin B, anisomycin, vancomycin, and bromocresol purple and bromothymol blue dyes know as PAV. The dyes color the *Legionella* colonies allowing easy phenotypic characterization and the antibiotics suppress competing flora
2. BCYE agar with polymymixin B, anisomycin, and cefamandole [12].

Additional pretreatment measures such as acid pre-treatment (HCL/KCL solution at pH 2.2) are often required to adequately inhibit respiratory flora. We would recommend acid pretreatment for sputum as a routine measure.

The specificity of culture approaches 99% [2, 10]. Recovering the isolate from culture also allows for detection of the source. The patient isolate and environmental isolate can be matched through molecular fingerprinting, such as pulsed field gel electrophoresis (PFGE) [6].

Culture results have major patient care implications since preventive measures can be enacted- either by disinfection of the water supply or expediting effective antibiotic therapy. A positive result usually appears within 3–5 days, although 2 weeks may be required because additional treatment may be necessary to reduce background flora that can inhibit the growth of *Legionella*. To avoid delays in diagnosis, we first perform direct culture (plate respiratory sample directly without pretreatment) and if overgrowth is observed after 3 days of incubation, Legionella Direct Fluorescent Antibody (DFA) staining of the specimen is performed followed by repeating the culture after acid pretreatment to reduce overgrowth [10, 13]. One drawback is that patients may have difficulty producing a suitable sputum specimen—a clinical characteristic traditionally associated with the “atypical” pneumonias. Given the high sensitivity and the implications for infection control, we recommend that all laboratories develop in-house capability for culturing using selective media. An impetus for more widespread use of respiratory tract cultures can be the presence of Legionella in the hospital water supply.

Environmental isolation requires different pretreatments techniques as well as different media. Culture media for environmental isolation requires addition of antimicrobial agents active against microbes found in environmental water. Two media are available commercially: (1) BCYE agar with bromocresol purple and bromothymol blue dyes, glycine, vancomycin, and polymyxin B called DGVP [14]. BCYE with cephalothin, coliston, vancomycin, and cycloheximide. This media is used primarily for culture water samples from cooling towers and other non-potable sites, as it contains antifungal agents [15].

## Direct fluorescent antigen

The direct fluorescent antibody (DFA) is a rapid test that requires expertise. The sensitivity of DFA is about 70% for detection of *L. pneumophila* serogroup 1 [11] with specificity approaching 99% [10, 13, 16]. DFA can be used as a confirmatory test for suspected *Legionella* colonies isolated from culture.

## Molecular tests

PCR and in situ hybridization have provided commercially available tools for a rapid diagnosis [17]. Commercially-available kits for PCR/RT-PCR for respiratory tract specimens have sensitivities ranging from 17 to 100% and specificities ranging from 95 to 100% [10, 13, 18, 19]. Genus probes and *L. pneumophila* probes have been developed, but results rarely identify specific species or serogroups. PCR can be performed in a few hours, but laboratory expertise is required. PCR assays for detection of Legionella in environmental water sources are commercially available. False positive results may exist when using PCR because molecular tests can detect non-culturable *Legionella* [20–22].

Loop-mediated isothermal amplification (LAMP) is a process similar to PCR, but requires less equipment, and shorter time for processing. Evaluation has been limited to environmental samples to date [23–26]. The specificity of the *L. pneumophila* species probe was 91% and a sensitivity of 100%, while the genus probe had a specificity of 93 and 100% when compared to the gold standard of culturing [26]. LAMP is less affected by inhibitory agents that would typically inhibit PCR results [26–28]. Given the advantages of rapidity of results, LAMP assays might be a supplement to culture. The cost per sample for LAMP is low relative to PCR because prior DNA extraction and thermal cycling equipment are unnecessary.

*Legionella* can be readily isolated from potable water systems. Non-potable sources include cooling towers and decorative fountains. Aerosolization was once thought to be the primary mode of transmission [29–31], but closer scrutiny shows that aspiration is the more common mode of transmission.

Environmental surveillance can increase the index of suspicion for hospital-acquired Legionnaires’ disease [32]. This is critical because Legionnaires’ disease is underdiagnosed and easily overlooked in hospital settings [33]. Few clinical laboratories have the resources or expertise to isolate *Legionella* from patient specimens.

Hospital hot water supplies can serve as a reservoir for other opportunistic pathogens, besides *Legionella* species [14, 34–36]. They include nontuberculous *Mycobacterium* spp. [37, 38], *Pseudomonas* spp. [39, 40], *Acinetobacter* spp. [41, 42], *Stenotrophomonas* spp. [43, 44], *Brevundimonas* spp. [45], *Sphingomonas* spp. [46, 47], and *Chryseobacterium* spp. [48]. These organisms can infect the same group of elderly and immunocompromised patients as *Legionella* and will likely be a growing area of concern as this patient population is increasing in number [1, 5].

## Conclusions

Physicians should adopt a proactive approach to diagnosis of pneumonias and anticipate the possibility of Legionnaires’ disease. Environmental surveillance and clinical surveillance of all nosocomial cases of pneumonia will lead to decreased morbidity and mortality. With the addition of efficient and accurate *Legionella* diagnostic laboratory testing, targeted antibacterial agent therapy can be administered.
